# Primary hepatoid adenocarcinoma of the lung in patient with silicosis: a case report and literature review

**DOI:** 10.3389/fonc.2024.1380717

**Published:** 2024-10-29

**Authors:** Lipeng Huang, Chaoyang Chen, Qingyu Sun, Zhichen Yu, Xiaoyan Wang, Xinle Wang, Shuoqi Yang, Luming Jin, Liang Bu

**Affiliations:** Department of Thoracic Surgery, Xiang’an Hospital of Xiamen University, Xiamen, Fujian, China

**Keywords:** hepatoid adenocarcinoma, lung malignancy, silicosis, lung metastasis, genetic testing

## Abstract

**Introduction:**

Hepatoid adenocarcinoma of the lung (HAL) is a special type of adenocarcinoma originating from the lung with adenoid- and hepatocyte-like differentiation. HAL is rare in clinical practice. Here, we present the case of a patient with HAL.

**Case presentation:**

A 59-year-old man was admitted to the hospital 4 days because of lung mas observed. Chest computed tomography (CT) revealed a lobulated mass shadow in the right lower lobe, approximately 3.5 × 3.3 cm in size. CT-guided percutaneous biopsy of the right lower lung was performed. The pathological results indicated a moderately to poorly differentiated adenocarcinoma. The patient underwent thoracoscopic right middle and lower lobectomy and systematic lymph node dissection. The postoperative pathology was primary HAL, with the staging of T2bN2M0 (stage III A). Recurrence-free survival and overall survival were 6 and 19 months, respectively Preoperatively, the level of alpha-fetoprotein was negative; however, after recurrence, it increased to 87.8.

**Conclusion:**

Pulmonary hepatoid adenocarcinoma is a rare subtype of malignant lung tumor, combined silicosis is more rare. Early surgical intervention can benefit patients in the early stages of the disease, whereas chemotherapy remains the main systemic treatment modality for postoperative and advanced stages. With the increasing popularity of genetic testing, it is important to focus on improving genetic examination.

## Introduction

Hepatoid adenocarcinoma (HAC) is a rare α-fetoprotein (AFP)-producing tumor with morphological similarities to hepatocellular carcinoma, characterized by eosinophilic cytoplasm, central nuclei, and tumor cells proliferating in a trabecular pattern, expressing AFP, HepPar1, HEA125, and MOC31 (1). In approximately 5% of HAC cases, the lung is the primary site of disease (1). HAC can metastasize to the brain, liver, adrenal glands, lymph nodes, and bones, with several cases reported. For patients with unresectable HAC, the prognosis is generally poor, with an overall survival of 6 to 11 months. This study reports a rare case of primary HAL complicated with silicosis and discusses the associated literature, including genetic information and comparisons with other case. A total of 94 cases of primary pulmonary hepatoid adenocarcinoma reported in July 2021 were collected from the literature. Another scholar collected 78 cases from the Surveillance, Epidemiology, and End Results (SEER) database for analysis and reporting.

## Case presentation

A 59-year-old man was admitted to the hospital because of “cough 4 days.” Chest computed tomography (CT) revealed a lobulated mass in the lower lobe of the right lung, approximately 3.5 × 3.3 cm in size. The mass had multiple burrs with rough edges, local bronchial truncation, CT value of approximately 48–58 HU on plain scan, and inhomogeneity after enhancement. The CT values were 90, 79, and 60 HU in the arterial, venous, and delayed phases, respectively. The patient had silicosis 20 years and smoking 30 years. CT-guided percutaneous biopsy of the right lower lung was performed before the operation. The pathological resulted adenocarcinoma. The immunohistochemical results of the tumor tissue were as follows: CK7 (partial +), TTF-1 (−), Ki-67 (about 30% +), napsin A (−), P504S (−), hepatocyte (partial +), Arg-1 (−), CK19 (+), CK20 (−), CDX-2 (+), p63 (−), Smur100 (−), GATA3 (small focus +), CK (+), CgA (−), Syn (−), and vimentin (−). According to the results of positron emission tomography–computed tomography (PET–CT), electronic gastroscope gastroscopy, electronic colonoscopy, and color Doppler ultrasonography of the hepatobiliary, pancreas, and spleen, we excluded the neoplastic lesions of other organs and considered the primary tumor of the lung. There were surgical indications and no obvious contraindications. The patient was treated with thoracoscopic right middle and lower lobectomy and systematic lymph node dissection. The postoperative specimens were sent for pathological examination.

**Figure 1:**
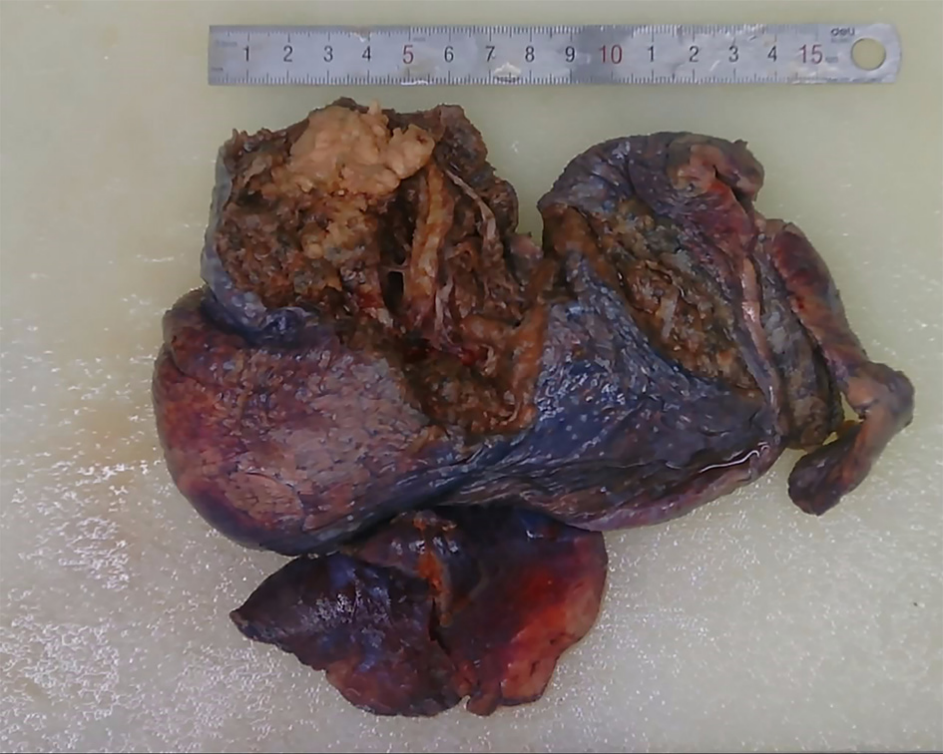
Specimen after surgery.

**Figure 2:**
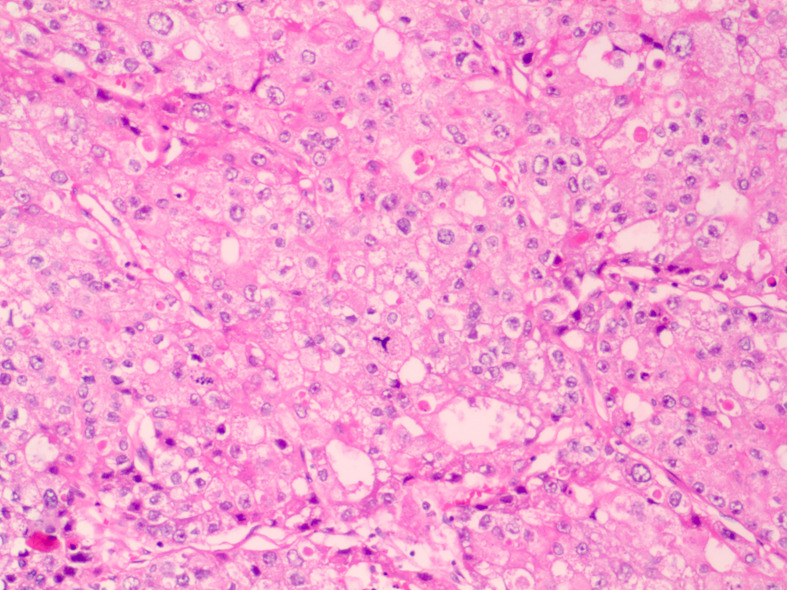
Pathological hematoxylin and eosin (HE) staining.

1. Gross specimen description: The lung lobe size was 16 × 10 × 3.5 cm; anastomotic nail length, 8 cm; bronchus broken end length, 1.2 cm; and tube diameter, 1.3 cm (**Figure 1**). The distance of the tumor from the pleura was 0.3 cm. The tumor that size 4.5 × 3 × 1.5 cm was from bronchial stump 3.5 cm, and no capsule, unclear boundary, gray–white section, and had medium texture.

2. Clinicopathology: The tumor cells showed eosinophilic cytoplasm, obvious nuclear atypia, pathological mitosis, necrosis, nesting, ethmoidal and tubular papilla (**Figure 2**). The diagnosis was as follows: hepatoid adenocarcinoma, tumor thrombus in the vessel, no cancer infiltration in the visceral pleura and bronchial incisal margin, and vitreous nodules in the parabronchial nodules. In addition, the group 7 lymph node was metastatic and the group 10 lymph node was normal. The level 2, 4, 8, 9, and 11 lymph nodes were found to have fibrous fat and necrotic vitreous tissue.

Tumor was not found on performing electronic colonoscopy and enteroscopy, and PET–CT examination before the operation did not show hypermetabolic shadow in the thyroid, liver, gallbladder, pancreas, stomach, or prostate. Hepatoid adenocarcinomas in the other organs were excluded and metastasized to the lung, which was diagnosed as primary HAL, with the clinical staging of pT2N2M0 (stage IIIA).

**Figure 3:**
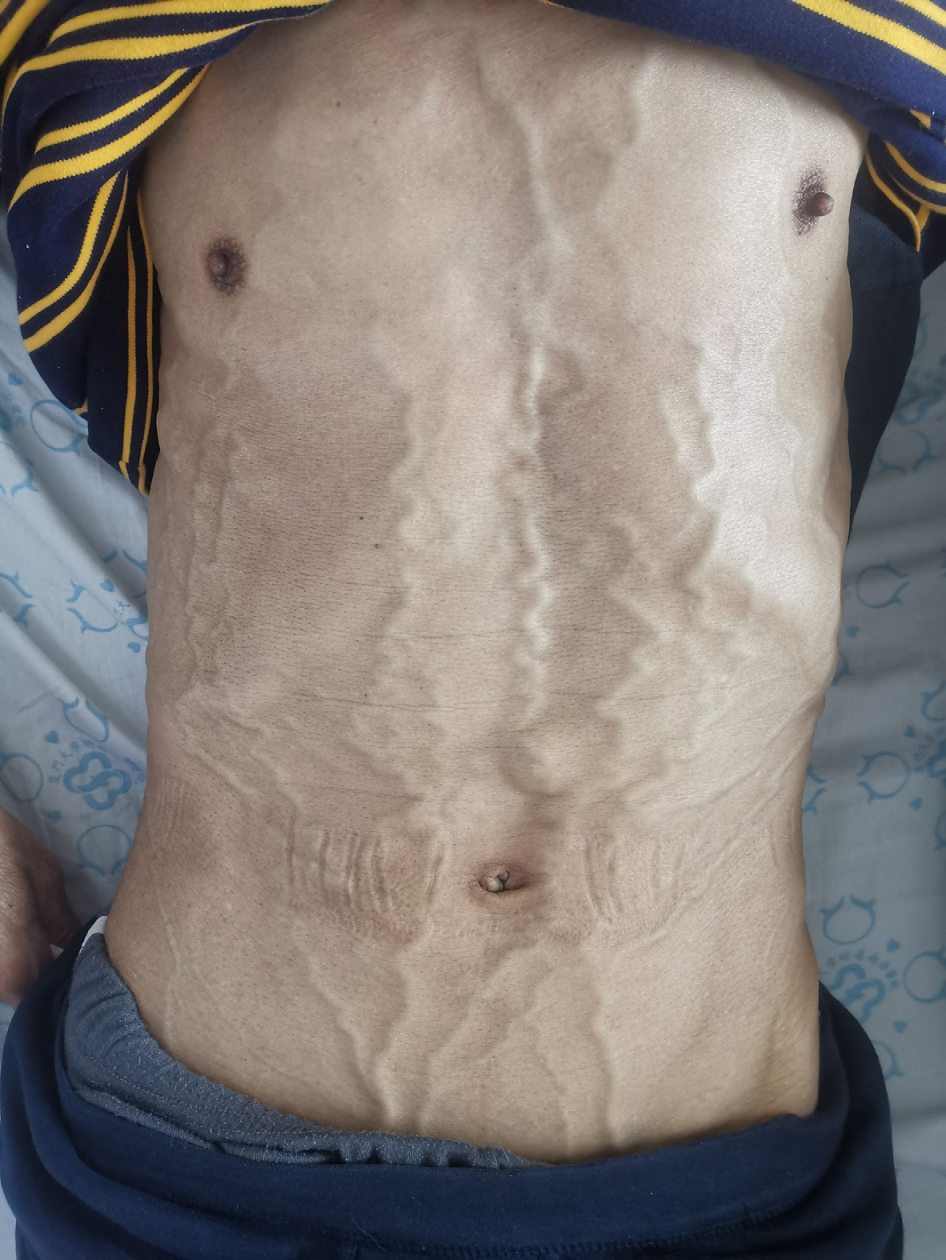
Secondary superficial venous varices in the patient after superior vena cava obstruction.

**Figure 4:**
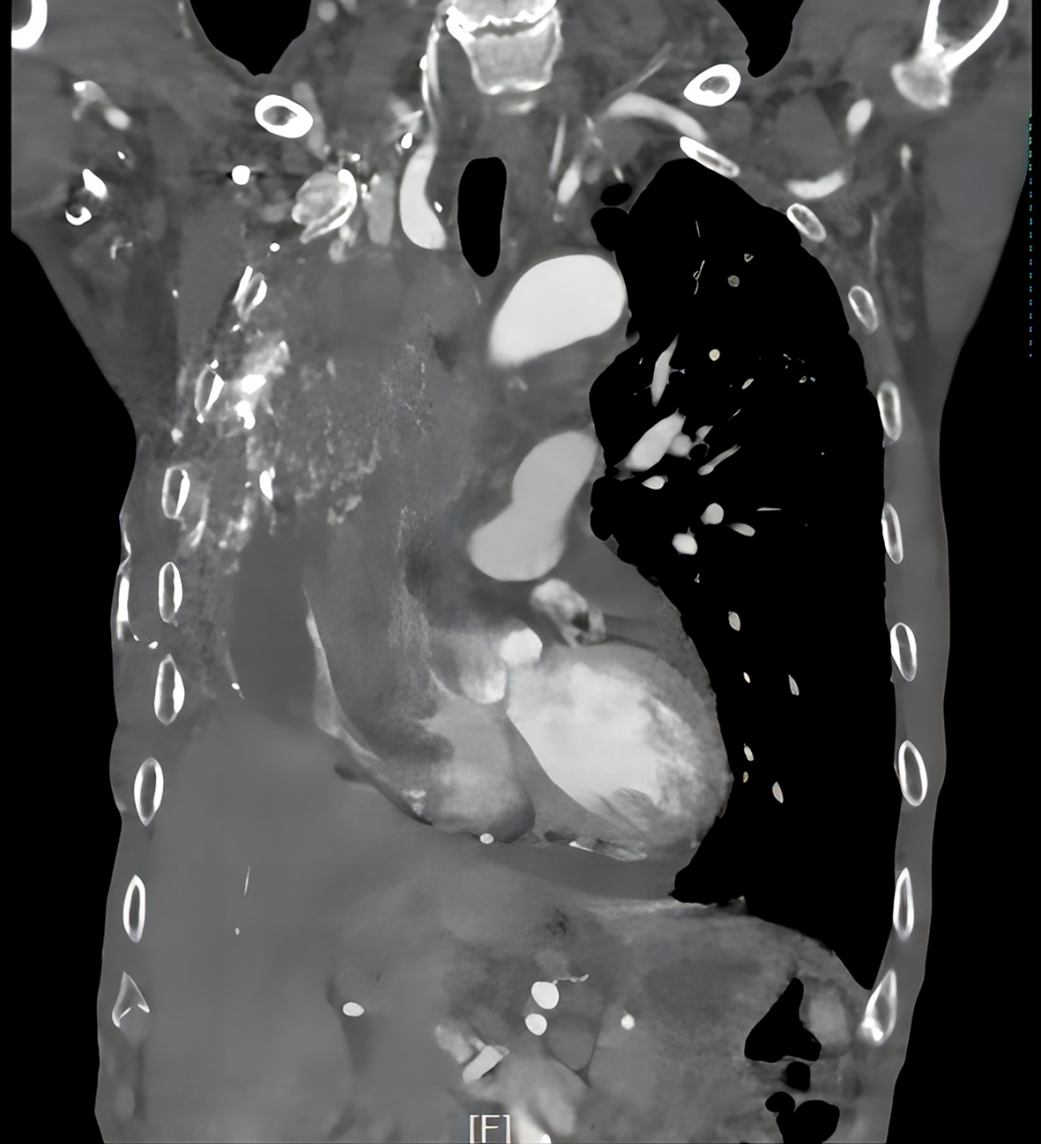
Tumor recurrence and invasion of the superior vena cava and right atrium.

3. Immunohistochemical results: CK7 (+), CK20 (focal focus +), villin (+), napsin A (spot focus +), TTF-1 (−), CDX-2 (partial +), CD56 (−), PSA (−), CEA (+), Ki-67 (about 40% +), hepatocyte (partial +), GPC-3 (partial +), Arg-1 (−), D2-40 (−), and CD30 (−).

4. Gene test results: gene: *STK11*, mutation: exon1 c. 232A > T p. K78 *, abundance: 28.10%; gene: *TP53*, variation: exon5 c.395A > T p.K132M, abundance: 30.45%; gene: *FANCI*, variation: exon21 c.2058G > T p.Q686H, abundance: 22.78%; gene: *FLT4*, variation: exon17 c.2413C > A p.H805N, abundance: 28.75%. Gene: *NOTCH1*, variation: exon9 c.1543_1548del p.E515_C516del, abundance: 27.68%; tumor microsatellite instability (MSI): microsatellite stable (MSS); tumor mutation load (TMB): 8.6667 mutations/mb.

5. Follow-up results: The patient underwent thoracoscopic right middle and lower lobectomy and systematic lymph node dissection on October 15, 2019. He refused postoperative adjuvant treatment. Owing to cervical lymph node enlargement, the patient underwent cervical lymphadenectomy, and primary HAL recurrence accompanied by lymph node metastasis was confirmed on April 2, 2021. At the same time, the patient had symptoms of hemoptysis and signs of varicose veins in the neck, chest wall, and abdominal wall (**Figure 3**). Chest CT revealed a mass (11 × 8 × 10 cm in size) in the right hilum of the lung, enlargement of multiple mediastinal lymph nodes, and tumor invasion in the superior vena cava and right atrium (**Figure 4**). These findings were considered to indicate tumor recurrence. However, no metastasis was observed in the liver, brain, bone, kidney, adrenal glands, or other organs. The alpha-fetoprotein (AFP) was 3.42 before the operation and 87.85 during the reexamination on March 12, 2021. The patient continued to refuse systemic antitumor therapy and eventually died on May 15, 2021.

## Discussion

Hepatoid adenocarcinoma is a special type of adenocarcinoma that occurs outside the liver at sites whose morphology is similar to that of hepatocytes. The most common sites include the stomach (63%), ovary (10%), lung (5%), gallbladder (4%), pancreas (4%), and uterus (4%) (15). Primary HAL is relatively rare. It was first proposed by Ishikura in 1990 (16). Also, the history of silicosis has not yet been reported. In this study, we have reported a case of primary HAL complicated with silicosis. The disease characterization is likely to improve the awareness on HAL.

1. Clinical case characteristics: The patient was admitted to the hospital for 4 days because of cough. Upon reviewing previous case reports, the clinical manifestations of HAL were found to be similar to those of common lung cancer, the symptoms of which include cough and expectoration, blood in the sputum, hemoptysis, and dyspnea. HAL may also be asymptomatic, depending on the location of the lesion and the stage of the disease. In this patient, the positive symptom was cough. Most patients with HAL have a history of smoking, and this patient also had smoking 30 years. In addition, he had a history of silicosis, which has not been reported in previous studies. Although the World Cancer Organization has identified quartz dust as a human carcinogen (17), there is no evidence to prove the correlation between silicosis and pulmonary hepatoid adenocarcinoma. The patient was found to have silicotic nodules in both lungs, which rendered our diagnosis and preoperative staging very difficult. We determined whether the nodules exhibited calcification on imaging and enhanced CT scan and also the F-fluorodeoxyglucose uptake value SUV_max_ on PET–CT. Based on the SUV_av_, the nodules were determined to be benign rather than tumor in the lung. The postoperative pathology confirmed the accuracy of our preoperative evaluation.
2. Pathological characteristics: According to the diagnostic criteria of pulmonary hepatoid adenocarcinoma proposed by Haninger et al. in 2013 (1), the tumor component could be simple hepatoid adenocarcinoma or hepatoid adenocarcinoma with typical acinar or papillary adenocarcinoma, signet ring cell, or neuroendocrine carcinoma (2); the AFP and other hepatocyte differentiation markers are not necessary, and (3) adenocarcinoma with the same morphological features as hepatocellular carcinoma but not producing AFP is called AFP-negative pulmonary hepatoid adenocarcinoma. This patients tumor was 4.5 × 3 × 1.5 cm in size, had ill-defined boundaries, no capsule, and gray–white section with medium texture. Microscopically, the tumors were arranged in nests, cribriforms, and tubular papillae. The pathology met the diagnostic criteria for hepatoid adenocarcinoma. The immunohistochemical characteristics were as follows: CK5/6 (1/5), CK7 (3/5), CK19 (4/5), CK20 (1/5), HEA125 (5/5), MOC31 (5/5), CEA (3/5), and napsin A (1/5). AFP is a sensitive marker for hepatocellular carcinoma but is not expressed in some pulmonary hepatoid adenocarcinomas. The results of tumor immunohistochemistry in this patient were as follows: CK7 (+), CK20 (focal +), villin (+), napsin A (spot +), TTF-1 (−), CDX-2 (partial +), CD56 (−), PSA (−), CEA (+), Ki-67 (approximately 40%+), hepatocyte (partial+), GPC-3 (partial+), Arg-1 (−), D2-40 (−), and CD30 (−). The preoperative serology of AFP was negative but became positive after reexamination on March 12, 2021.
3. With the continuous in-depth research on targeted therapy and immunotherapy, the detection of tumor genes has become an important basis for treatment. Thus, we sequenced tumor-related genes in the pathological tissue of this patient, and the abnormally mutated genes detected were *STK11, TP53, FANCI, FLT4*, and *NOTCH1*. The tumor was of the MSS type, and the tumor mutational burden (TMB) was 8.6667 mutations/mb. The immunotherapy-related PD-1/PD-L1 gene was not expressed. Although we failed to detect clinical targeted therapy or immunotherapy-related mutation sites in this case, we further analyzed and explained the detected mutated genes in combination with previous studies. *STK11* is a tumor suppressor gene that encodes serine–threonine kinase 11 (LKB1) (18).According to the COSMIC database, the rate of STK11 gene mutation in lung adenocarcinoma samples was 7.6% (246/3235). In the present case, the mutation was nonsense mutation in exon 1 of the STK11 gene, which caused the original codon encoding lysine to become a stop codon. This mutation was predicted to induce protein function inactivation. Gene mutations were also observed in the cases reported by Sunhui et al. and accounted for 11% (2/17) of the cases reported in the literature (**Table 1**).

**Table 1:** Previously reported cases on hepatoid adenocarcinoma of the lung (HAL) containing genetic information.

No.	Author	Year	Sex	Age	Smoking	Staging	Treatment	Gene	Immunohistochemistry	Metastasis	Prognosis(month)
1	Haninger (1)	2014	M	65	+	pT2aN0M1b(IVA)	Surgery+Chemotherapy	EGFR(−)	N/A	N/A	10
2	GavrancicT (2)	2015	M	64	−	CT2aN2M1(IV)	Chemotherapy+Targeted therapy	EGFR(−)	AFP(+),HepPar-1(+),CK7(+),Napsin-A(+),TTF-1(+),CK5(−),CK6(−),CK20(−).	Spinal,rib,lymphnode	11
3	Grossman Kate (3)	2016	M	54	+	cT3N0M1b(IVA)	Chemotherapy	ALK(−),EGFR(−),K-ras(−)	AHepPar-1(+),CK7(+),CAM5.2(CK8/18)(+),CEA(+),AFP(−),CK20(−),P63(−),CDX2(−),PSA(−),S-100(−),CgA(−),TTF-1(−)	Brain	3
4	BasseV (4)	2018	M	43	+	cTxN3M1c(IVB)	Chemotherapy+Immunitytherapy	EGFR(−),KRAS(−),ALK(−),ROS1(−),a loss of expression of mutL homolog 1 and PMS1 homolog 2	CK7(+),CK19(+),CK20(+),HepPar-1(+),CEA(+),TTF-1(+);PD-L1(−)	Brain,bone,adrenalglands	N/A
5	Barbara CD (5)	2019	M	63	N/A	N/A	Chemotherapy+Immunity therapy	EGFR(−),ALK(−)	N/A	Skin	7
6	Chen HF (6)	2019	M	53	–	pT3N0M0(IIB)	Surgery+Chemotherapy+Targeted therapy	EGFR p.L747_P753delinsS;EGFR exon 20 T790M mutation	ATTF-1(+),HepPar-1(+),AFP(+),CK(+),napsin A(−),CK5/6(−),P63(−),CD56(−),Syn(−)	N/A	36
7	EI Khoury A (7)	2019	M	50	+	cT4N2M1b(IVA)	Chemotherapy	EGFR(−),ALK(−),BRAFw/t.ROS-1(−),PDL1≥50%	CK7(+),HepPar-1(+),TTF1(−),CK5/6(−)	N/A	14
8	Kuan K (8)	2019	M	47	+	cT4N0M0(IIIA)	Surgery	EGFR(−),ALK(−),PDL1(high)	CK7(+),EMA(+),CEA(+),HepPar-1(+),TTF-1(−),napsin A(−),CK20(−),AFP(−)	N/A	N/A
9	Li J (9)	2019	M	71	–	cT3N3M1b(IVA)	Radiotherapy	EGFR,ALK,ROS1,PDL1,BRAF,HER2,KRAS,MET,RETWildtype;FAT1-Mutated,Copynumber loss;MSI;TMB-1.69mutations/mb	CK(+),SALL-4(+),CK18(+),CK8(+),CK7(+),AFP(+),HepPar-1(+),STAT-6(+),CD117(+),CK20(−),P63(−),P40(−),CK5/6(−),Syn(−),CD56(−),CgA(−),Vim(−),calretinin(−),TTF-1(−),napsin A(−),CD34(−),D240(−),ALK(−),PD-L1(−)	Bone	5.5
10	Wang C (10)	2019	M	70	+	cT3N2M0(IIIB)	Chemotherapy+Immunity therapy	TP53 mutation	HepPar-1(+),CK(+),EA(+),CDX-2(+),AFP(−),TTF-1(−),NapsinA(−),P63(−),P40(−),CD56(−),Syn(−)	N/A	9
11	WangXP (11)	2019	M	48	+	cT4N3M1a(IVA)	Chemotherapy	EGFR(−),ALK(−),ROS1(−),KRAS(−),BRAF(−)	AFP(+),HepPar-1(+),Arg-1(+),P40(−),CK5/6(−),P63(−),TTF1(−),ALK(−),CgA(−),Syn(−),CD56(−),PGP9.5(−),napsin A(−)	bone	12
12	Chen LL (12)	2020	F	65	–	pT4NxM1b(IVA)	Chemotherapy+Targetedtherapy+Immunitytherapy	ALK(−),EGFR(−),KRAS hotspot G12V(c.35G>T)mutation	ASALL4(+),AFP(+),GPC3(+),CK7(+),villin(+),TTF(−),napsin A(−),HepPar-1(−)	N/A	52
13	Wang W (13)	2020	M	41	–	N/A	Radiotherapy	EGFR and KRAS(−)	CK(+),CK7(+),PAS-AB(+),vimentin(+),hepatocyte(+),napsin A(−),TTF-1(−),Ki-67(60%),CD56(−),CD31(−),desmin(−),ERG(−),HMB-45v,melan A(−),MiTF(−),S-100(−),SMA(−),STAT6(−)	N/A	12
14	Sunhui (14)	2021	M	66	+	N/A	Surgery	TP53 SMARCA4CDK8 EPHA5	CKpan(+);CK18(+);CK7(−);CK20(−);TTF1(−);napsin A(−);CGA(−);Syn(−);hepatocyte(−);AFP(+);glypican-3(+);SALL-4(+);SMARCA4(+)	N/A	46
15	Sunhui (14)	2021	M	64	–	N/A	Surgery	TP53 CDKN2A STK11 INPP4B	CKpan(+);CK18(+);CK7(+);CK20 (–)TTF1(−);napsin A(−);CGA(v);Syn(−);hepatocyte(+);AFP(−);glypican-3(−);SALL-4(+);SMARCA4(+)	N/A	26
16	Sunhui (14)	2021	M	64	–	N/A	Surgery	TP53 SMARCA4 CDK8 EPHA5	CKpan(+);CK18(+);CK7(+);CK20(−);TTF1(−);napsin A(+);CGA(−);Syn(−); hepatocyte(+);AFP(+);glypican-3(−);SALL-4(+);SMARCA4(−)	N/A	23
17	Present case		M	59	+	pT2N2M0(IIIA)	Surgery	STK11,TP53,FANCI,FLT4,NOTCH1	CK7(+),CK20(+),villin(+),napsin A(+),TTF-1(−),CDX-2(+),CD56(−),PSA(−),CEA(+), Ki-67(40%+),hepatocyte(+),GPC-3(+),Arg-1(−), D2-40(−),CD30(−)	lymphnode	19

The tumor suppressor gene *TP53* was the most frequently mutated gene in pulmonary hepatoid adenocarcinoma, accounting for 29.4% (5/17). The COSMIC database reported that the mutation rate of the *TP53* gene in lung adenocarcinoma samples was 31.46% (973/3093). In the present case, the mutation was a missense mutation in exon 5 of the *TP53* gene, which was predicted to induce inactivation of the encoded protein.

*FANCI* encodes a FANC protein involved in DNA replication and DNA damage response, and certain deletion mutants are associated with increased risk of various cancers (19, 20). The COSMIC database reported that the mutation rate of the *FANCI* gene in lung adenocarcinoma samples was 0.8% (9/1124). The mutation was identified as a missense mutation in exon 21 of the *FANCI* gene. Nevertheless, the effect of this mutation on protein function has not yet been documented. The proto-oncogene *FLT4* encodes FMS-like tyrosine kinase-4, also known as vascular endothelial growth factor receptor 3 (Vegfr-3). Vascular endothelial growth factor C (Vegf-C) signaling via Flt-4 promotes lung adenocarcinoma invasion and metastasis of cancer cells (21). The COSMIC database reported that the mutation rate of the *FLT4* gene in lung adenocarcinoma samples was 2.84% (39/1375). The mutation was identified as a missense mutation in exon 17 of the *FLT4* gene. However, the effect of this mutation on protein function has not yet been reported.

*NOTCH1* regulates cell differentiation, growth, proliferation, survival, and metabolism from embryonic development to adulthood (22). *NOTCH1* amplification or high protein expression is correlated with the tumor stage, grade, size, metastasis, and venous invasion (23, 24). The COSMIC database reported that the mutation rate of the *NOTCH1* gene in lung adenocarcinoma samples was 2.21% (44/1987). The mutation was identified as noncoding deletion mutation in exon 9 of the *NOTCH1* gene. At present, the effect of this mutation on the protein function is unknown, but the tumor has a strong ability for venous invasion, which appears to be related to this gene mutation. Immunotherapy was FDA-approved for the treatment of microsatellite instability-high or deficient mismatch repair solid tumors that have progressed during initial therapy or have no replacement therapy. The TMB detected in this case was 8.6667 mutation/mb, which is a low level, thereby suggesting that the effect is not ideal.

Another unusual phenomenon was that approximately 50% of conventional lung adenocarcinoma cases had somatic mutations in genes encoding proteins of the EGFR signaling pathway, such as K-RAS, EGFR, HER2, HER4, BRAF, and pik3ca (25). However, only one case reported by ChenHF et al. was positive for EGFR mutation; the rest were negative (16/17), the proportion of which was far lower than that of lung adenocarcinoma.

Based on the results of genetic testing, we did not find any sensitive genes for lung targeted therapy and immunotherapy, thus suggesting that this case may not be sensitive to the aforementioned therapies.

By reviewing previous case reports, we found that surgical resection was the main treatment for stage I–III pulmonary hepatoid adenocarcinoma, and platinum-based dual systemic antitumor therapy was the main treatment for stage IV. The patient in this case underwent thoracoscopic right middle and lower pulmonary resection and systematic lymph node dissection following complete preoperative examination. He was found to have cervical lymphadenopathy 6 months after the surgery and thus underwent superclavian lymph node resection. The patient was diagnosed with metastatic poorly differentiated carcinoma after the surgery, which indicated recurrence of pulmonary hepatoid adenocarcinoma with lymph node metastasis. Liang Zhenzhen et al. (26) searched the SEER database and identified 78 cases of HAL in the United States from 2001 to 2016 for statistical analysis. Only six patients received surgical treatment, which was less than that reported in the literature.

4. Prognosis and follow-up: According to literature reports, the 1-, 2-, and 5-year overall survival (OS) rates of 68 patients were 29.38%, 14.39%, and 5.88%, respectively, and the median survival time was 5 months (26). In another research, the 1-, 3-, and 5-year OS rates of 94 patients were 37%, 30%, and 15% for men and 63%, 63%, and 38% for women, respectively (27). Because most of the cases were reported at present, the statistical difference was large. The 1-year OS rates of patients who underwent surgery- and chemotherapy-based treatment strategies were 53% and 30%, respectively (27). The patient in this study only underwent surgical treatment. The postoperative stage was T2bN2M0 (stage III A), and the recurrence-free survival was 6 months. Six months after the operation, the patient underwent cervical lymphadenectomy owing to cervical lymphadenopathy, and the OS was 19 months. CheYQ et al. reported that their case showed extensive venous invasion. It was controlled by radiotherapy and chemotherapy, but then progressed and spread (28), which was very similar to this case. After recurrence in this patient, the tumor mainly invaded the lymph nodes and veins. In addition, it invaded the brachiocephalic and innominate veins as well as superior vena cava and extended down to the right atrium, occupying the right. A large amount of space in the atrium and severe signs of superior vena cava stenosis, manifested by jugular venous distention and extensive superficial trunk varicose veins. The patient eventually died 19 months after the operation. We considered two main causes of death: one was advanced tumor cachexia and the other was that the tumor invaded the right atrium and continued to progress and led to thrombosis, thus resulting in further mechanical obstruction.

In summary, pulmonary hepatoid adenocarcinoma is a rare subtype of malignant lung tumor, with few reported cases and no large-scale clinical trials; thus, to date, there are no standardized diagnosis and treatment plan. Early surgical intervention can benefit patients in the early stages of the disease, whereas chemotherapy remains the main systemic treatment modality for postoperative and advanced stages. With the increasing popularity of genetic testing, it is important to focus on improving genetic examination. If specific tumor-driving genes are identified through genetic testing, corresponding targeted therapies can be considered. Furthermore, it is expected that more genetic mutations of pulmonary hepatoid adenocarcinoma can be analyzed to provide an evidence for guiding chemotherapy, targeted therapy, and immunotherapy.

## Data Availability

The raw data supporting the conclusions of this article will be made available by the authors, without undue reservation.
